# Improved parenting maintained four years following a brief parent training intervention in a non-clinical sample

**DOI:** 10.1186/s40359-016-0150-3

**Published:** 2016-08-24

**Authors:** Charlotte Reedtz, Sihu Klest

**Affiliations:** Center for Child and Youth Mental Health & Child Welfare, Faculty of Health Sciences, UiT-Arctic University of Norway, Tromsø, Norway

**Keywords:** Parent training, Parenting, Child behaviour problems, Universal prevention, Incredible years, Community services

## Abstract

**Background:**

The aim of the present study is to evaluate whether the effects of a short, six session version of an evidence-based parent training programme (The Incredible Years), delivered in a non-clinical community sample in the northern Norway, are maintained 4 years following the initial intervention.

**Method:**

Data were collected primarily from mothers in a randomized controlled trial (*N* = 117). Children’s mean age at 4 year follow-up was 7.5 years.

**Results:**

A mixed model analyses of linear change with a time by condition interaction revealed that statistically significant differences were maintained between the parent training and control groups for several outcomes. The parent training group showed a reduction in harsh disciple and an increase of both self-reported positive parenting and parental efficacy when compared to the control group who received services as usual. No significant differences between the two groups were found for child behaviour problems as measured by the ECBI Intensity scale. In addition, mixed model analyses of quadratic change were conducted to test the differences in the trajectory of change over four time points. There were significant differences in the trajectory of change for (1) the ECBI with the parent training group showing an immediate drop in the intensity of problem behaviour and (2) the positive parenting scale showing an immediate steep increase; no other significant differences in trajectory were detected.

**Conclusions:**

Families from a non-clinical sample who participated in a brief version of the Incredible Years Basic parent training programme maintained changes in positive parenting, harsh discipline, and parental efficacy 4 years after completion of the intervention.

**Trial registration:**

ClinicalTrials. gov NCT02850510. Retrospectively registered 29 July 2016.

## Background

The aim of the present study is to evaluate whether the effects of a short, six session version of an evidence-based parent training programme (The Incredible Years), delivered in a non-clinical community sample in the northern Norway, are maintained 4 years following the initial intervention. As a result of the findings of this study Carolyn Webster-Stratton has developed a new programme in the Incredible Years series called “Attentive Parenting” for universal populations.

A substantial body of research indicates that parenting practices that provide children with positive non-disciplinary interactions, enthusiastic play where the adult follows the interest of the child, positive attention, and a responsive, sensitive, and nurturing environment contributes to promoting good child mental health and well-being and preventing the development of socio-emotional and behavioural problems (e.g., [1–5]). Conversely, dysfunctional family interactions, such as harsh and inconsistent parenting, are significant risk factors for child maltreatment and poor socio-emotional and behavioural development in children (e.g., [3, 6–9]). While parenting quality is a powerful influence on the development of maladaptive behaviour, it is also a modifiable risk factor [4, 10].

In addition to the devastating effects behaviour problems can create for families, there is considerable financial burden to society associated with problem behaviour in children. One study conducted in England found that children who had been assessed with conduct disorder at age 10 had cost social service agencies 10 times more by the time they reached 28 years than controls without conduct disorders [11]. These human and financial costs will likely not decrease without intervention, as time trends of problems in childhood and adolescence has generally found that emotional and behavioural problems have either significantly increased or remained the same over the past five decades [12].

In a review of the literature on evidence-based psychosocial treatments for child and adolescent disruptive behaviours, Eyberg, Nelson, and Boggs [13] conclude that Parent Traning (PT) should be the preferred approach for treating young children. Many other researchers and child service organizations have come to the same conclusion. A meta-analysis of preventive parenting interventions found strong evidence for the effectiveness of these programmes [14]. The National Institute for Health and Clinical Excellence in the U.K. recommends the early use of PT interventions to prevent antisocial personality disorder later in life [15], and the World Health Organization concluded that parenting programmes can prevent violence against children and have the potential to prevent children from perpetrating violence later in life [16]. PT programmes have been shown to be the most effective treatments for families of preschool children with conduct problems [17, 18]. Early parental intervention has also been found to counter biological and environmental risk factors in children, creating a more positive developmental trajectory than would be expected without intervention [10, 19]. The most effective treatment programs available are those based on social learning principles and social interaction learning theory [20, 21], and these programs highlight parents’ role as children’s interactive partners, instructors and providers of social activities and opportunities for their children. Or as stated by Haslam, Mejia, Sanders & de Vries (2016), [21] “Parenting programs are interventions that aim to improve child and family outcomes by equipping parents with effective parenting skills”. Our understanding of how parents influence the development of disruptive behaviour problems owes much to the work of Gerald Patterson and his colleagues [22]. There is now overwhelming evidence that inadequate parental monitoring and parenting practices characterized by high levels of harsh and inconsistent discipline predicts the development of antisocial behaviour both in childhood and in adolescence [22]. Their theory of “coercive family processes” has been one of the most influential approaches in understanding the development of behaviour problems in childhood.

Programmes such as the Incredible Years (IY) teach effective parenting skills, including how to encourage appropriate behaviour, enhance play and interact in a supportive manner with the child, as well as to employ more positive discipline techniques while reducing harsh and negative parenting [20]. These aims can help to increase parents’ sense of competence in the skills they use with their children [23]. Parenting competence is related to parenting self-esteem, a concept that integrate both self-efficacy as a parent, as well as the satisfaction parents get from parenting [24]. Parenting competence typically refers to the degree to which parents feel competent in dealing with child problems. Moreover, parental sense of efficacy is proposed by Sandler, et. al [14]. to be a potential mechanism for the positive outcomes observed with PT; they indicate that research is lacking for the long-term effects of prevention programs effects on parenting skills and perceptions of parental efficacy. In a study of families with children in the Head Start programme, Mendez-Baldwin, and Busch-Rossnagel [23] found that improving parental sense of competence may have contributed to more positive and less negative parent–child interactions. Similarly, Ohan, Leung, and Johnston [25] found that externalizing child behaviour problems were negatively correlated with parents’ reports of satisfaction with their parenting role.

Although the body of literature supporting the effectiveness of evidence-based parenting programmes is growing [4], and a substantial number of quality studies have established the effectiveness of PT programmes for high risk groups (e.g., [6, 13, 18]), few studies have investigated the long-term effects of brief parenting programmes in non-clinical and non-high risk community samples beyond 2 years post intervention [26–30]. One study on the IY Programme that has followed a selective community sample failed to find sustained effects of the intervention [27]. In this study, Scott and colleagues tested the effects of the full scale IY programme with children aged 4–6 years at high risk of developing antisocial behaviour; the children had elevated, but not clinical, scores for problem behaviour and lived in a deprived area of London. The intervention showed moderate effects immediately following completion the programme, however, none of the effects were sustained when families were assessed an average of 5.8 years after completing the intervention. This finding is in contrast to a parallel study reported in the same paper [27] that showed sustained results an average of 7.8 years following treatment with the IY programme in a clinical sample of young children who had been referred to services for their problem behaviour. In this sample, favourable effects were found for oppositional defiant symptoms, antisocial personality traits, parenting skills, and child reading skills. In their discussion of the differences between the selective and indicated study groups, the authors conjecture that parents may not have been as motivated to keep up their positive parenting practices in the selective group because their children’s problem behaviour was not as severe as the indicated sample. As these studies are some of the only long-term research conducted with Incredible Years programmes, in particular with a non-clinical sample, further investigation of the effects of these programmes with non-clinical community samples is called for. Currently, those with without a clinical diagnosis often do not receive help until their problems have progressed [31], resulting in distress and turmoil for the families as well as the possibility of poorer outcomes in the future. In addition, reaching families before severe problems develop may reduce the resources that are needed to keep serious issues at bay and allow for a larger percentage of the population to benefit from services. The RE-AIM model illustrates this issue and posits that public health cannot be changed if an intervention only reaches a small percentage of the population, but that less resource intensive interventions may allow for changes that can be seen on a population level by reaching more of those who require assistance [32]. Similarly, Kazdin and Blase assert that although many advances have been made in the effectiveness of individual psychotherapy, the need for these services is so great that it is currently not possible to provide the necessary care to those in need and that alternative delivery methods are necessary to reach a larger population of those who could benefit from the current knowledge in the field [33].

The present study addresses the research gap in brief programmes with the potential for large scale implementation by testing a shortened version of the IY Basic parenting programme (S-IY) in a non-clinical community sample 4 years after completion of the intervention. The IY parenting programme was used because it has been implemented throughout Norway, and has an existing framework for successful implementation with many trained practitioners. This brief intervention, primarily focuses on enhancing prosocial behaviours and positive parent–child interactions and was developed to add to the public services of mental health promotion in families with young children. An RCT of this S-IY found a significant reduction in self-reported harsh parenting and child behaviour problems, as well as enhancement of self-reported positive parenting and of the parents’ sense of competence for the group who received the S-IY programme, as compared with the control group who received services as usual [30]. With the exception of child problem behaviour and parental sense of efficacy, these effects were maintained at 1-year follow-up, although with a reduction in effect sizes. The purpose of the present study is to conduct follow-up assessments with these families 4 years after completion of the initial intervention to determine whether effects were maintained or changed for (1) parents’ levels of self-reported positive and harsh parenting, (2) parents’ sense of competence, as measured by efficacy and satisfaction, and (3) child behaviour problems.

## Method

### Participants

A total of 269 families volunteered to participate in the study, which took place in the largest city in the northern part of Norway with a population of about 75,000 people. The study population is generally representative of families in the city, region and country where it was conducted [34]. Most families in this sample had mothers working full time (61 %) and were two-parent families (80 %) with one or two children (79 %). For more information, see Reedtz et al. [34].

Children who scored above the 90^th^ percentile on the Eyberg Child Behavior Inventory (ECBI) Intensity scale were excluded from the study; in total 58 children (22 %) whose parents agreed to participate scored above this cut-off. These families were offered the full 12–14 week Incredible Years Basic programme to ensure that they received sufficient treatment. Of the remaining 211 families a total of 22 families (10 %) terminated participation before the programme started.

Based on ECBI scores reported by primarily mothers, 189 children between 2 and 8 years met the inclusion criteria for this study. Although children’s ECBI scores appeared to be somewhat higher than the mean scores of a Norwegian sample reporting stratified means [35], they were still within the normal range. Both the mother and father responded in 112 cases (59 %), only the mother responded in 74 cases (39 %), and only the father responded in 3 cases (2 %). Mothers and fathers mean age at baseline was 35 and 37 years respectively.

Families were randomly assigned to either the intervention (*n* = 92) or the control group (*n* = 97) at baseline. At baseline, the intervention and control groups were similar in demographic characteristics with no statistically significant differences between the two groups. Demographics are reported in Tables 1, the majority of families at baseline and 4 year follow-up were two-parent families, had completed a bachelor degree or higher, and worked full time. Analyses of families who dropped out of the study at 4 year follow-up revealed that there were no statistically significant differences between families who completed the final assessment and those who did not on both demographic variables and all measures used in assessments. This was true both for analyses comparing the families who dropped out (*n* = 49) to those who remained in the study (*n* = 65), as well as for analyses examining differential dropout between the control and intervention groups. The following baseline variables were included in these analyses and no statistically significant differences were found: (1) Mother’s education level, (2) mother’s age, (3) mother’s work status, (4) mother’s marital/cohabiting status, (5) number of children, (6) PPI Positive Parenting, (7) PPI Harsh Disciple, (8) PSOC Efficacy, (9) PSOC Satisfaction, and (10) ECBI Intensity Scale.

**Table 1:** Parent demographic characteristics

	Baseline		4 year follow-up	
	S-IY	Control group	S-IY	Control group
Mother				
College Education^a^	80.4 %	75 %	82.3 %	85.7 %
Employed Full-time	59.8 %	62.9 %	74.2 %	89.2 %
Married/Cohabiting	84.8 %	76.3 %	85.5 %	69.4 %
Father				
College Education	66.7 %	66.7 %	81 %	79 %
Employed Full-time	90 %	90.7 %	78 %	70 %
Married/Cohabiting	91.7 %	98.1 %	94.6 %	100 %

^a^Bachelors degree (or equivalent) or higher

At baseline 112 boys (59 %) and 77 girls (41 %) were enrolled in the study; child age ranged from 2 to 8 years, with a mean age of almost 4 years. At 4 year follow-up the mean child age was 7.5 years and a similar sample make-up was maintained with 66 boys (58 %) and 48 girls (42 %).

**Fig. 1:**
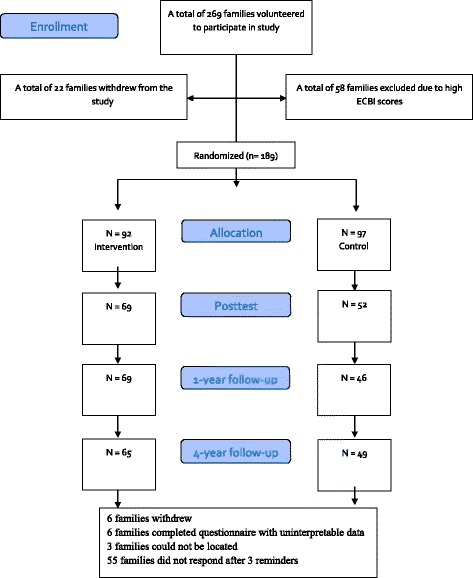
Consort diagram of participants from baseline to 4 year follow-up

The response rates for post-test, 1 year follow-up, and 4 year follow-up were 75.3 %, 75.3 % and 73 % respectively for the intervention group and 53.6 %, 47.4 %, and 51 % for the control group. At 4 year follow-up 111 mothers and 60 fathers completed questionnaires. Both the mother and father responded in 57 cases (50 %), only the mother responded in 54 cases (47 %), and only the father responded in 3 cases (3 %). Because fewer fathers responded, only the data from mothers were used in analyses, with the exception of the three families where only the father responded. In this case, the fathers’ reports were used to include all children in the study. However, the term mothers will be used, because the analyses are predominantly conducted using mothers’ responses. A consort diagram illustrates families’ research participation over the 4 years (Fig. 1).

### Measures

Parents were asked to complete questionnaires at pre, post, 1 year follow-up, and 4 year follow-up. Both mothers and fathers were given four measures (1) a family demographics questionnaire (e.g., number of siblings the target child has, marital status, employment status, and education), (2) Parenting Practices Interview, (3) Parenting Sense of Competence, and (4) ECBI.

#### *Parenting Practices Interview* [36]

The Parenting Practices Interview (PPI) was adapted from the Discipline Questionnaire that was developed at Oregon Social Learning Center, and research has demonstrated adequate reliability and validity when used with parents of young children [36]. Two subscales were used: Positive Parenting (α = .67) and Harsh Discipline (α = .79). The subscale *Positive Parenting* is 15 items detailing the respondents’ parenting methods and the frequency with which they praise, reinforce, and reward their children (e.g., “During an ordinary week, how often do you praise or reward your child for good behaviour at home or at school?”). The subscale *Harsh Discipline* is 14 items detailing parents’ disciplinary practices including parents use of force through verbal and physical aggression (e.g., “If your child behaves in a negative way, how probable is it that you would spank the child?”). All items are rated on a 7-point scale (*1 = never* to *7 = always, or 1 = not probable* to *7 = very probable*).

#### *Parenting Sense of Competence* (PSOC) [24]

The PSOC is a 16-item measure intended to assess parents’ beliefs that they are capable of doing a good job parenting their child. It is comprised of two subscales and is rated on a 6-point scale from *1 = strongly agree* to *6 = strongly disagree*. The efficacy subscale measures parents perceived competency (e.g., “Being a parent is manageable, and my problems are easily solved”), while the satisfaction subscale measures parental satisfaction (e.g., “Being a parent makes me tense and anxious”). Research on the PSOC has demonstrated adequate reliability and validity when used with parents of young children [24], and the reliability scores for these subscales in the present study at 4 year follow-up were α = .69 and α = .77 respectively.

#### *Eyberg Child Behavior Inventory* (ECBI) [37]

The ECBI is a 36-item parent report measure designed to assess child behaviour problems (e.g., “is overactive or restless”, “lies”, and “hits parents”). Internal consistency at 4 year follow-up was α = .82. Parents rate problem behaviour on two dimensions, the frequency of the behaviour (from *1 = never, to 7 = always*) and identification of the behaviour as a problem for the parent (*yes* or *no*). Only the parents’ ratings of the frequency of problem behaviours, the Intensity subscale, was analysed in the present study. The ECBI is the only measure that has been standardized in Norway to assess conduct problems in children aged 2 to 17 years [35].

### Procedure and design

The study was conducted in one municipality in Norway, where all parents of children between the ages of 3 and 5 years (3,000 families) received an invitation through the mail to participate in a short parenting programme aimed at preventing child development of socio-emotional and behaviour problems. In addition, families with children between 2 and 8 years of age were recruited through posters in day care, kindergartens, schools, and advertisements in local newspapers. Parents contacted the university in their city to enrol in the study, at which time the procedures were briefly explained. Parents who agreed to participate were asked to complete the inventories described above and return them in a pre-paid envelope together with a signed letter of informed consent. All participants agreed to answer the same questionnaires several times in the following 10 years. If there was more than one child between 2 and 8 years in the household, the youngest was selected as the target child in the study. The study was approved by the Regional Committee for Ethics in Medical Research at the University of Tromsø.

When parents agreed to participate they were randomised to receive the S-IY programme or to receive the usual services that were available to them in the community. The measures were completed at four time points: before and after the intervention (pre- and post), and 1- and 4 years after the intervention (1 year follow-up and 4 year follow-up). All families, regardless of condition assignment, completed the questionnaires on the same time schedule.

#### The intervention

The intervention group participated in a shortened version of Webster-Stratton’s “Basic Parent Programme” from the Incredible Years programme series. This version covered the first six meetings in the original manual of the programme. This shortened version was approved by the programme developer Carolyn Webster-Stratton. Groups of parents for 6 to 8 children met for 6 sessions held once weekly at a local health care centre. Both mothers and fathers were invited to participate. Each of the two hour sessions was led by two Incredible Years trained group leaders. In this version of the programme, parents were taught positive disciplinary strategies (play, praise, and rewards) through group discussion, role-play, home practice activities, and watching Incredible Years video vignettes during the groups. The excluded content was related to principles of ignoring negative behaviour, effective limit setting and time out.

#### Group leaders

A total of 15 health nurses trained in specialised public health care administered the S-IY groups. All had experience in clinical work and were trained in the IY programme according to procedures established by the programme developer. The group leaders received continuous supervision through observations, role-play, and video reviews from a certified IY trainer and two certified IY mentors. The mentors and trainers were also certified according to procedures established by the programme developer.

#### Intervention integrity

The group leaders followed the standard IY treatment manual for the first six sessions, completed standard check-lists after each session, and tracked group activities (number of video vignettes shown, role-plays, and parent tasks between sessions). All group sessions were videotaped for evaluation by an IY mentor. Select tapes were reviewed with the S-IY group leaders at weekly peer and self-evaluation meetings.

#### Four year follow-up

Four years following completion of the intervention, participants were asked to complete the same battery of questionnaires. The measures were sent to all participants in the study by mail with a pre-paid return envelope and a letter detailing the follow-up study. Only three families from the original cohort could not be located. In total, 114 families returned completed questionnaires.

### Analytic approach

A mixed model analysis of linear change [38] with a time by condition interaction was conducted to test the differences between the families who received the S-IY intervention and the control group 4 years post intervention. This method controls for participants’ pre-intervention scores.

In addition, a mixed models analysis of quadratic change was conducted to test the differences in the trajectory of change over the four time points (pre, post, 1 year follow-up, and 4 year follow-up) between the S-IY group and the control group.

Effects sizes were calculated using Hedges *g* [39]. Cohen [40] describes a small effect for Hedges *g* to be 0.20, a medium effect to be 0.50, and a large effect 0.80 or greater.

We used Intention to Treat analysis (ITT) for all outcomes to include every family who completed any part of the first assessment regardless of when they may have dropped out of the study. Full information Maximum Likelihood (FIML) was used to estimate the parameters in the model. This method allows for the inclusion of individuals with missing data and introduces one of the lowest levels of bias to the data set due to missing data [41]. Means and standard deviations for family characteristics and outcomes are also reported.

Version 22 of the statistical software package SPSS [42] was used to conduct all analyses apart from the power analyses which were computed by hand.

## Results

Means, standard deviations, and group differences from pre to post, pre to 1 year follow-up, and pre to 4 year follow-up are presented in Table 2.

**Table 2:** Means, standard deviations, and group differences from pre to post, pre to 1 year follow-up, and pre to 4 year follow-up

	Incredible Years				Control Group						
	Pre	Post	1 year FU	4 year FU	Pre	Post	1 year FU	4 year FU	Pre-post	Pre-1 year	Pre-4 year
	M	M	M	M	M	M	M	M	t_(383)_	t_(386)_	t_(385)_
	(SD)	(SD)	(SD)	(SD)	(SD)	(SD)	(SD)	(SD)	(g)	(g)	(g)
PPI Positive Parenting	4.56	5.20	5.06	4.93	4.50	4.52	4.69	4.50	7.04***	3.72***	3.80***
	(0.49)	(0.54)	(0.47)	(0.44)	(0.56)	(0.62)	(0.63)	(0.64)	(1.13)	(0.62)	(0.63)
PPI Harsh Discipline	1.96	1.70	1.74	1.74	1.93	1.90	1.87	1.84	4.12***	−2.65**	−2.63**
	(0.48)	(0.44)	(0.43)	(0.41)	(0.38)	(0.44)	(0.43)	(0.41)	(0.57)	(0.38)	(0.37)
PSOC Efficacy	31.38	33.94	34.24	34.35	31.93	32.63	33.56	33.27	3.13**	1.90	2.67**
	(3.64)	(3.62)	(3.56)	(3.86)	(3.69)	(3.80)	(3.93)	(4.30)	(0.28)	(0.18)	(0.25)
PSOC Satisfaction	39.43	42.73	43.42	43.92	40.24	41.30	42.00	42.76	2.66**	2.36*	1.54
	(6.56)	(6.44)	(5.90)	(5.44)	(5.88)	(5.77)	(6.25)	(5.86)	(0.59)	(0.54)	(0.35)
ECBI Intensity	104.25	96.10	96.01	92.81	101.91	102.44	99.18	89.06	−2.75**	−1.74	0.303
	(18.59)	(18.40)	(21.59)	(21.27)	(14.50)	(19.39)	(18.53)	(17.69)	(0.48)	(0.32)	(−0.05)

*p < .05, **p < .01, ***p < .001

### Positive parenting scale (PPI)

#### Change

At 4 year post intervention, mixed model analysis with a group by time interaction revealed that the significant difference between the S-IY and control groups was maintained from previous time points for the PPI Positive Parenting measure, with the S-IY group scoring higher on self-reported positive parenting than the control group *t*(383) = 3.80, *p* = 0.001, *g* = 0.63. The results indicate that the intervention had a medium to large effect on scores on Positive Parenting.

#### Trajectory

**Fig. 2:**
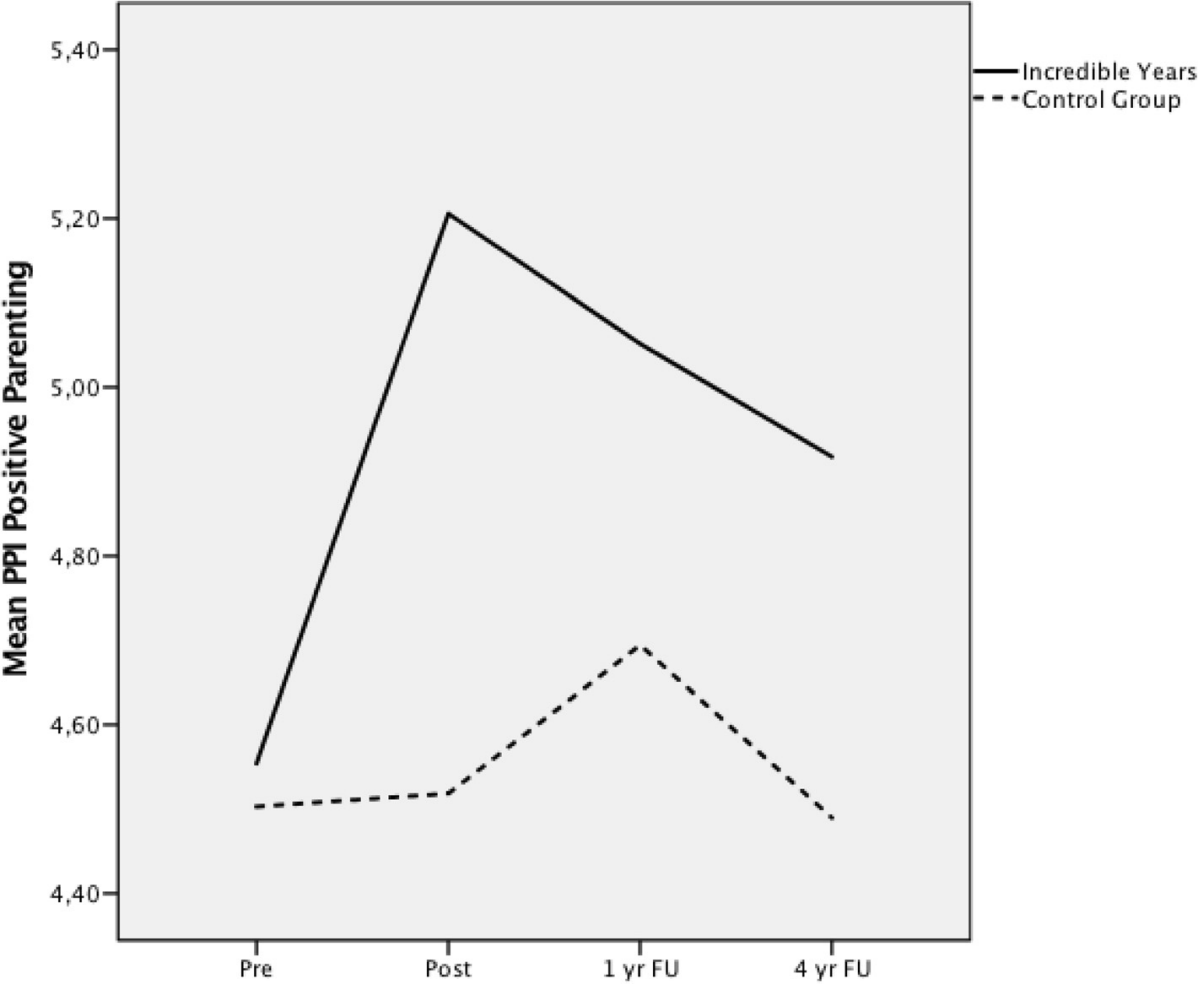
Growth trajectory for PPI Positive Parenting scale over four time points

There was also a significant difference in the trajectory of change over the time points between the groups, *F*(1, 271) = 4.50, *p* = 0.04 (Fig. 2). The group who received the S-IY program showed an immediate steep increase in self-reported Positive Parenting that declined somewhat in the following years, but remained significantly higher than at pre-intervention. The control group changed at a slower rate and at 4 year follow-up had maintained the same levels of self-reported Positive Parenting as at pre-intervention (Fig. 2).

### Harsh discipline (PPI)

#### Change

The S-IY group showed a significant drop on the PPI Harsh Discipline scale from pre to 4 year follow-up when compared to the control group, *t*(370) = −2.63, *p* = .01, *g* = 0.37. The results indicate that the intervention had a small to medium effect on scores on Harsh Discipline.

#### Trajectory

**Fig. 3:**
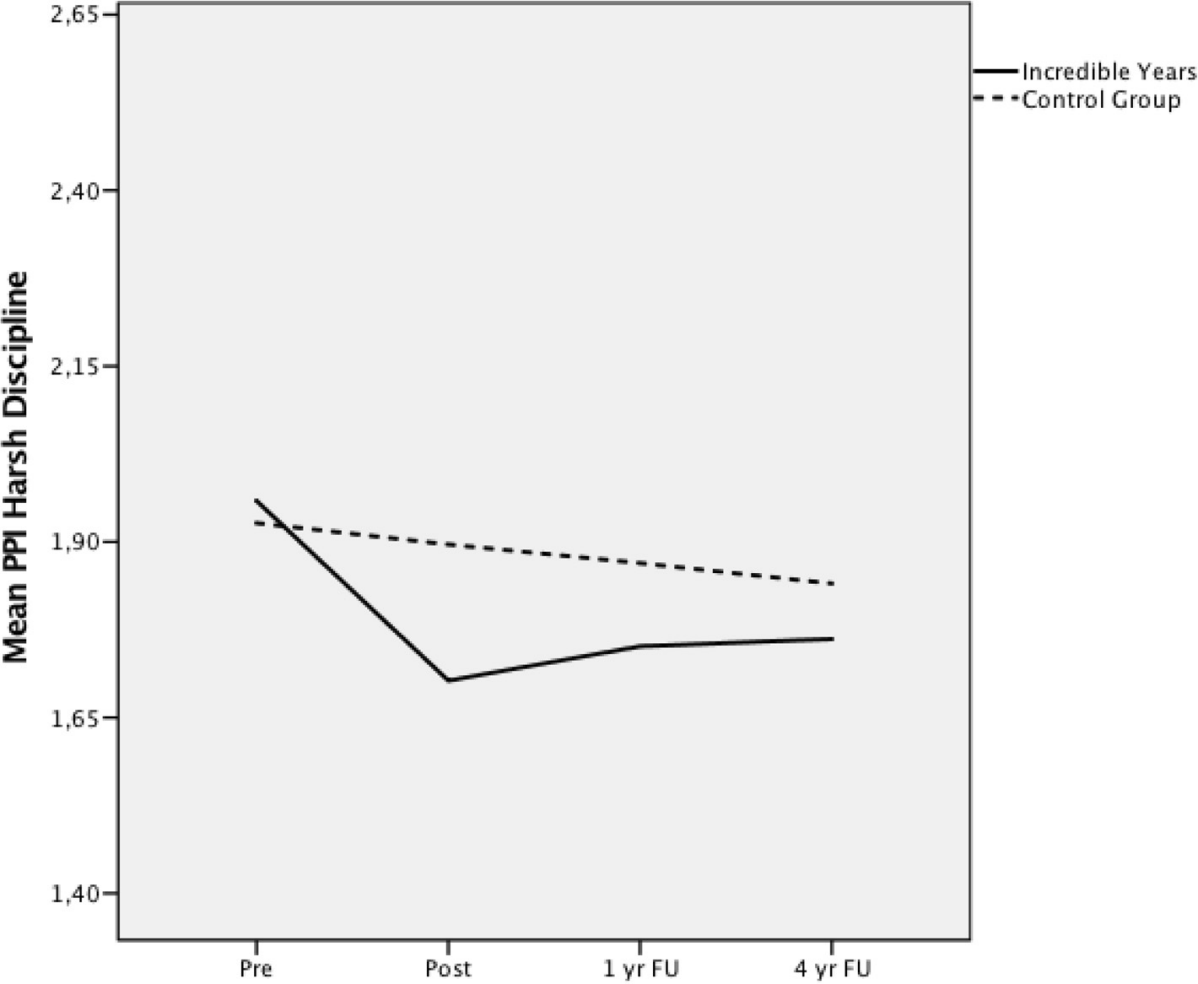
Growth trajectory for PPI Harsh Discipline scale over four time points

There was no difference in the trajectory of change between the groups, *F*(1, 263) = 2.91, *p* = .09 (Fig. 3).

### Parental efficacy (PSOC)

#### Change

The S-IY group scored significantly higher on parental efficacy than the control group from pre to 4 year follow-up as measured by the PSOC Efficacy scale, *t*(372) = 2.67, *p* = 0.01, *g* = 0.25. The results indicate that the intervention had a small effect on scores on Parental Efficacy.

#### Trajectory

**Fig. 4:**
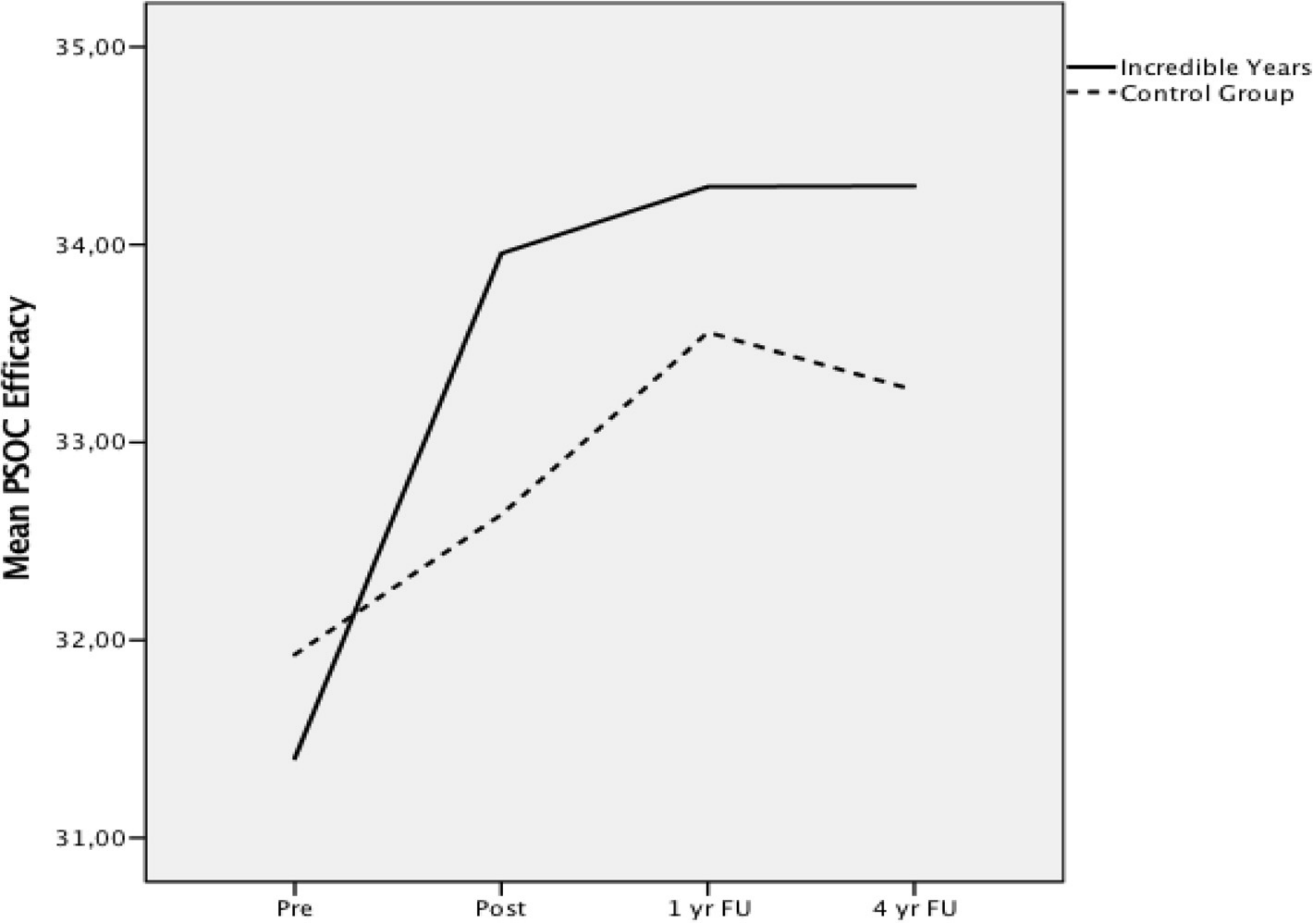
Growth trajectory for PSOC Parenting Efficacy scale over four time points

The two groups did not show a difference in the trajectory of change over time, *F*(1, 257) = 0.84, *p* = 0.36 (Fig. 4).

### Parental satisfaction (PSOC)

#### Change

The improvement that was seen for the S-IY group on the PSOC Satisfaction scale at 1 year follow-up was no longer statistically significant 4 years after the intervention, *t*(360) = 1.54, *p* = 0.13, *g* = 0.35.

#### Trajectory

**Fig. 5:**
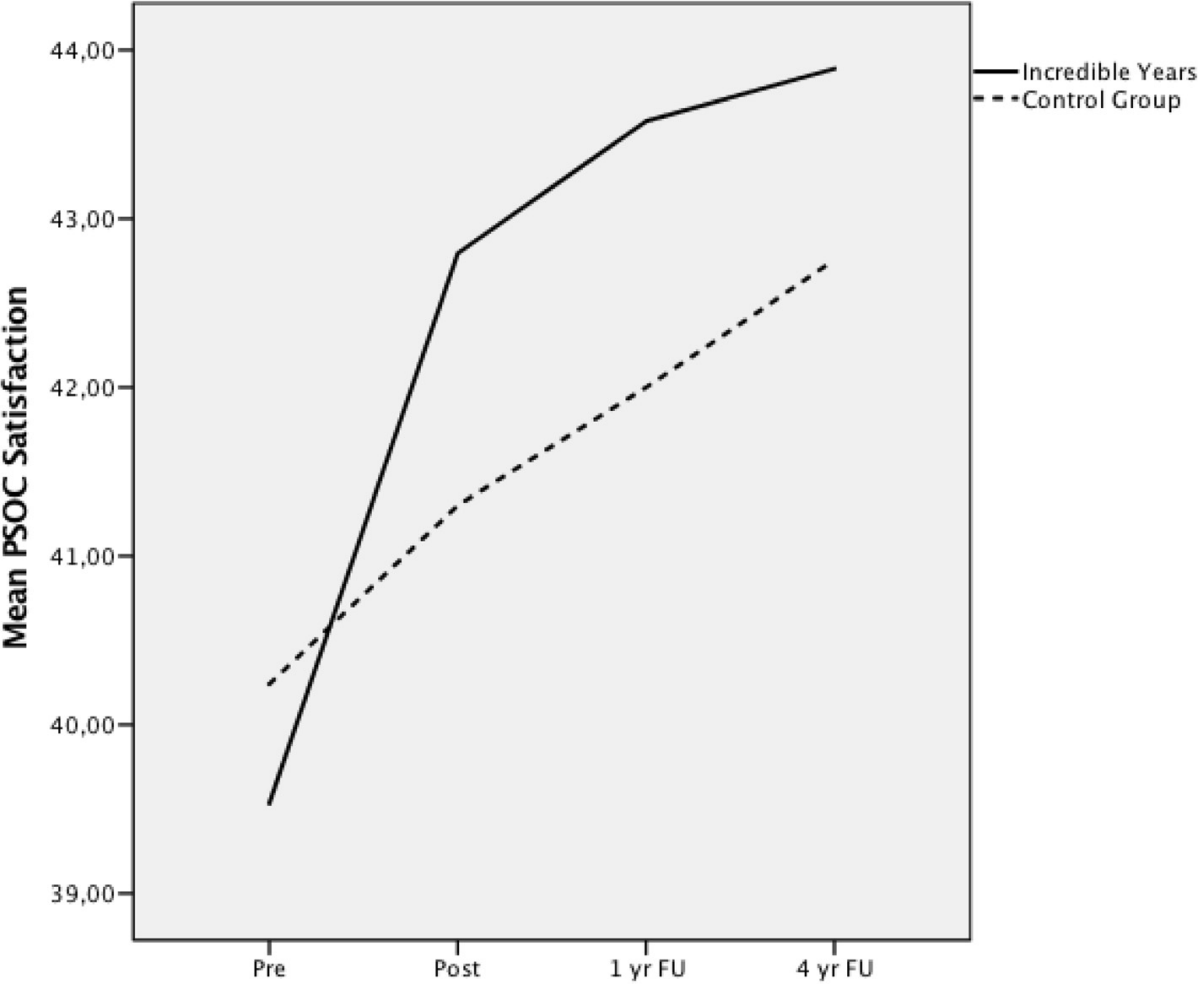
Growth trajectory for PSOC Parenting Satisfaction scale over four time points

The trajectory of change was also not statistically significant when comparing the two groups, *F*(1, 254) = 3.17, *p* = 0.08 (Fig. 5).

### Child behaviour problems (ECBI intensity)

#### Change

There was no statistically significant difference between the intervention and the control groups on the intensity of child behaviour problems at 4 year follow-up, *t*(385) = 0.30, *p* = 0.76, *g* = − 0.05.

#### Trajectory

**Fig. 6:**
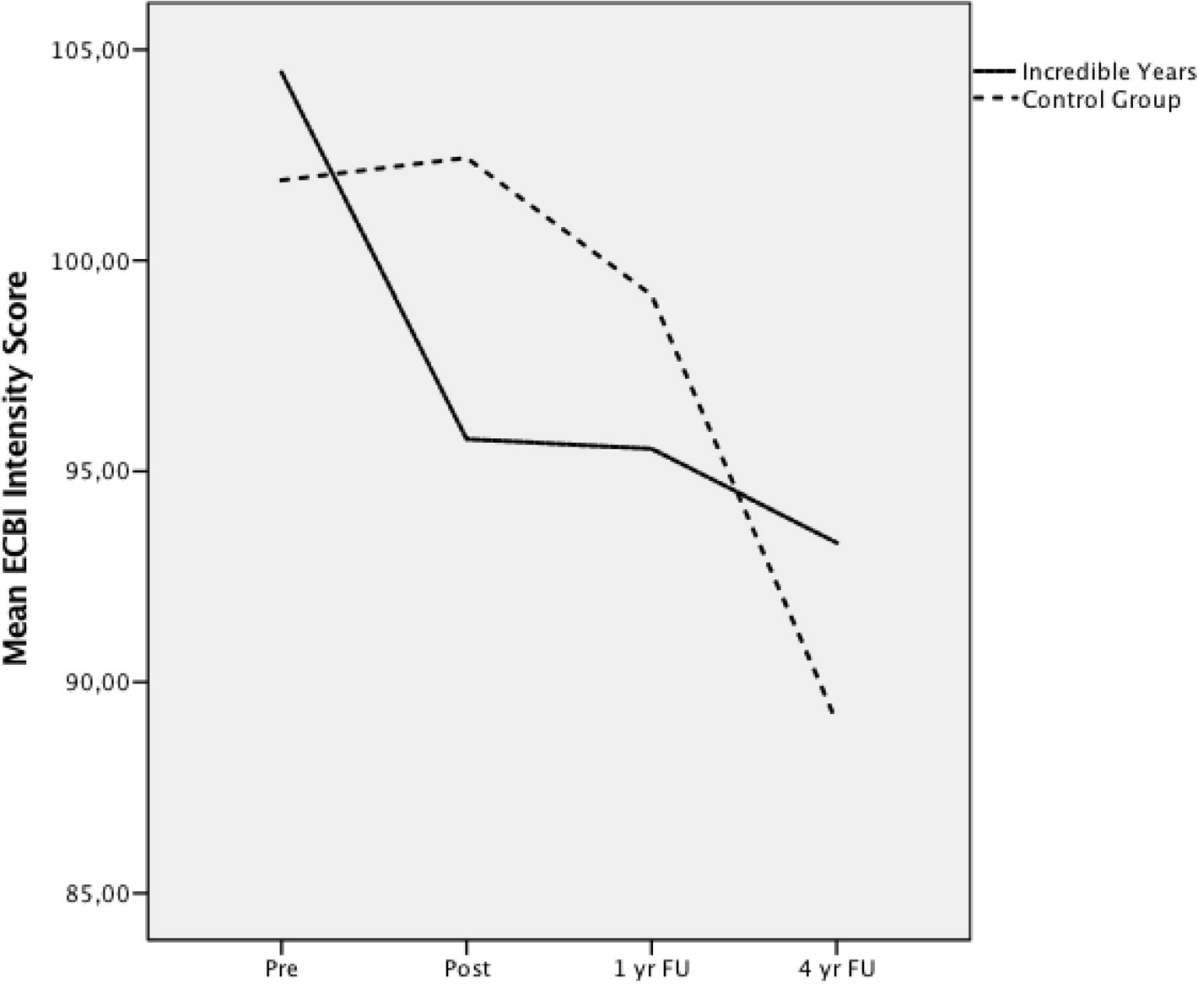
Growth trajectory for ECBI Behaviour Intensity scale over four time points

Although no differences on ECBI intensity scores were found, growth curve analysis revealed a significant difference in the trajectory of change for the two groups, *F*(1, 265) = 4.33, *p* = .039. The group who received the S-IY showed an immediate drop in the intensity of child problem behaviour following the intervention. This significant reduction levelled off and remained stable through the 4 year follow-up assessment. In contrast, the control group, who received services as usual, maintained a higher level of child problem behaviour at post intervention and showed a slower decline until the levels matched those of the intervention group at 4 year follow-up (Fig. 6).

## Discussion

In the initial RCT of the S-IY Basic programme, Reedtz et al. [30] found that the S-IY group scored significantly higher than the control group on self-reported positive parenting practices (PPI), parental efficacy and satisfaction (PSOC), and significantly lower on harsh discipline (PPI) and child behaviour problems (ECBI). The changes pre- to post-intervention on mothers’ ratings of self-efficacy and child behaviour problems were not maintained at 1 year follow-up, however, all improvements in self-reported parenting practices were maintained. These findings from a brief preventive and health promoting parent intervention for a non-clinical sample achieved some of the central goals as the full version of the Incredible Years programme; improved parenting skills and reduced negative parenting strategies.

In the present 4-year follow-up study, mothers maintained significantly increased self-reported positive parenting (medium to large effect), a reduction in harsh discipline (medium effect), and an increased sense of efficacy (small effect). There were no significant differences between the groups on parental sense of satisfaction or child behaviour problems.

Considering the substantial body of research that supports positive parenting as one of the most important predictors of pro-social child behaviour [17, 20, 43] and well-being [44], this S-IY programme has the potential to contribute to the promotion of pro-social child behaviours and sound socio-emotional development in young children. The differences between the S-IY and control groups on parenting practices suggests that mothers in the intervention group use positive parenting strategies such as, problem-solving, giving the child opportunities to correct mistakes, praising the child, giving compliments, offering privileges, tokens and reinforcements for positive behaviours, as well as kissing and hugging the child, significantly more often than the mothers in the control group. The effect size of the increased positive parenting strategies is medium to large and the trajectory of change shows that the S-IY programme results in an immediate steep increase in such parenting behaviours compared to the control group. A boosted investment in the parent–child relationship at around 4 years of age, aided by this type of early PT, may serve as an example of efforts to, “increase the prevalence of nurturing environments”, as Biglan and his colleagues [44] highlight in a recent review.

Considering the substantial body of research showing that dysfunctional family interactions are important predictors of child maltreatment and child problem behaviour [7, 9, 10, 45], this S-IY programme has the potential to reduce harsh parenting strategies. The mothers in the intervention group dropped significantly from pre to 4 year follow-up compared to the control group in the use of negative parenting strategies such as raising their voices and yelling at the child, threats to punish the child physically or non-physically, grounding the child, hitting the child, flicking the child’s ear, as well as slapping the child. The control group also reduced their use of such strategies, but at a slower pace, and to a lesser degree. A possible explanation for the gradual reduction in the use of harsh parenting strategies in the control group is that problem behaviours naturally decline as children grow older in a universal population [35]. Considering these results, it seems likely that parents in the intervention group learned new parenting skills through their participation in the S-IY programme and that these skills were maintained 4 years following the end of the S-IY programme; it is possible that this change was maintained through positive responses from their child and strengthened parent–child interaction.

Scores on parents’ sense of competence showed a significant increase in parental efficacy (small effect) from pre to 4 year follow-up for the mothers in the S-IY group. The higher level of perceived efficacy in the S-IY group is stable from post-intervention to 4 year follow-up, while it continuously decreased in the control group beginning at 1 year follow-up. Previous research has suggested that in universal community samples, where the level of child problems are generally low, parents, and mothers in particular, may not use this absence of problems as a basis by which to judge their effectiveness [25]. Rather, they may rely on other aspects of child behaviour, such as child academic achievements, as well as their use of high levels of nurturance, support for their children in achieving normative developmental tasks, and their use of effective control strategies [14]. Gardner et al. [20] tested the effectiveness of a parenting intervention delivered in a community-based organisation and found, in contrast to previous studies [23],that changes in parents’ sense of competence did not contribute to child outcome, whereas change in positive parenting skills did. Based on the results in the present study it is possible that parents’ perception of efficacy in the parenting role is positively related to aspects of positive parenting. There is some research available to demonstrate the causal relationship between parents perceived efficacy and general parenting behaviours [46], and several empirical papers report on associations between effective parenting strategies and lower child mental health problems [14].

The significant difference between the groups that was present at 1 year follow-up on the measure of parental sense of satisfaction, was not maintained at 4 year follow-up. There is a trend toward this result in the present study which may indicate that if more families had been included in the study a significant difference may have been found. Previous studies have reported stronger effects of PT on parents’ sense of competence (both on subscales on parental efficacy and parental satisfaction) [25], however, these were parents of clinically referred children. The natural decline in problem behaviour as children grow older a universal populations may serve as an explanation of the rise in parental satisfaction for both groups throughout the study.

Child behaviour problems were reduced immediately following the S-IY programme, however, this effect was small and not maintained one and 4 years after the intervention. The Basic Incredible Years programme has been shown to significantly reduce problem behaviour among children in a number of studies [20, 36, 47], both short term and long term. However, these studies have used clinical samples with children diagnosed with oppositional defiant and conduct disorders, where the potential for reduction of problem behaviour is larger than in sample beginning in the normal range child behaviour. The current sample is a non-clinical and non-high risk community sample, where children with elevated ECBI scores were excluded. Because the children were all in the normal range of behaviour, we did not expect large changes as a result of the S-IY programme. In addition, problem behaviour naturally declines with increasing age, resulting in a negative correlation between ECBI scores and age [35]. This may be the reason for the gradual decline in ECBI scores over time for both groups. However, the intervention group showed a large drop initially, and then declined at smaller increments during follow-up assessments. As this group has little room for improvement past posttest scores, the potential for reduction in ECBI scores are larger in the control group. In other words, this result likely indicates that the intervention group reached the natural low point for problem behaviour more quickly than the control group.

As dysfunctional parenting is not only related to child behaviour problems, but a wide range of health, social, and educational difficulties in children and young people, Sanders et al. [48] proposes a population approach that seeks to improve parental competency on a larger scale. With the addition of the shortened version of the Basic Incredible Years programme, the intervention that best reflects the families’ specific needs can be used, in line with the principle of minimal sufficiency. This brief parenting intervention requires low practitioner and parent time commitment, but can have a significant impact on parenting practices and sense of competence. In addition, the limited scale of this S-IY programme makes it cost effective for broad implementation at community level. A study on the costs of a public health infrastructure for delivering parenting and family support [8] concludes that it is quite feasible to implement population-wide efficacious parenting programmes aimed at reducing child behavioural and emotional problems and promoting effective parenting. Their estimates suggest that delivery costs could be recovered in a single year if they support as little as a 10 % reduction in the rate of child abuse and neglect. This S-IY programme may fit well into a population level approach to family support.

### Limitations and future directions

One important limitation of the present study that we only examined child behaviour based on parents’ perceptions. Parents’ reports of child behaviour may be biased and not representative of the child’s true behaviour. There is evidence to suggest a correlation between parent self-report measures and observation scores [49], however, including direct observation and multiple reporters would strengthen the results by eliminating self-report bias.

The relatively low internal consistency with Cronbach’s alphas at .67 on the Positive Parenting subscale of the PPI, and at .69 on the efficacy subscale of PSOC indicates measurement error, but this would have worked against our hypothesis.

Families with children who had high ECBI scores, and have a potential for greater change, were excluded from this study, therefore, this sample is not a true universal population. In addition, our sample was obtained through self-referral and the demographic make-up was generally homogenous with somewhat higher than average socioeconomic status. Most of the participants were two-parent families (80 %), worked full time (61 %), and had obtained a high level of education (49 % held Masters or Ph.D. degrees). Reyno and McGrath [50] found that low education or occupation status predicted only moderate treatment effects for PT, while families with higher levels of social resources showed stronger treatment effects. In contrast, demographic variables, such as maternal age, maternal level of education, and single-parent family status have not had an effect on treatment outcomes for populations in the U.S. [51], United Kingdom [52], and Norway [53]. The somewhat skewed make-up of this population may have affected the outcomes, but it is not clear in what direction. Further research in to demographic make-up should be done to ensure that the programme is used in settings where it is most effective.

Analyses are based on the responses primarily from mothers with the inclusion of three fathers who were the sole respondents. Acquiring sufficient data from fathers would strengthen the results in that the potential for differential effects of the programme for mothers and fathers could be tested.

There was participant attrition from the first assessment to 4 year follow-up. Although we used ITT and FIML analyses, participation rate may reduce the validity of the results. The higher level of dropout in the control group is likely a result of parents’ lack of motivation to complete an extensive battery of questionnaires when they did not receive any additional services.

## Conclusions

The results indicate that the S-IY programme enhances self-reported positive parenting and parental sense of efficacy while decreasing harsh discipline in a non-clinical and non-high risk community sample at least 4 years after completion of the intervention. Our hope is that future research will evaluate whether enhancement of effective parenting may create a more positive developmental trajectory for children and whether brief parenting interventions may be a useful tool in community services, particularly given the low level of resources required from both practitioners and parents to participate in such programmes. In addition, future research should evaluate whether brief parenting interventions have the potential to create the necessary conditions for children’s healthy development and to promote well-being, as well as to prevent the long term development of socio-emotional and behavioural problems. For those at greatest risk, the strongest effect of parent training interventions may be a reduction in potent risk factors; such as violence, threats, and other harsh discipline, while for those at lower risk, the most important effect of the same intervention may be to strengthen protective factors such as the increased use of positive parenting techniques [54].

## Abbreviations

ECBI: Eyberg Child Behavior Inventory
PPI: Parenting Practices Interview
PSOC: Parents Sense of Competence
PT: Parent Training
RCT: Randomized Controlled Trial
S-IY: Short Incredible Years Programme
